# Progress in Surgery

**Published:** 1895-12-07

**Authors:** 


### Progress in Surgery.

### GENERAL SURGERY.

**Fractures and Dislocations** (*continued from page* 151).—Experience teaches that the sooner after a sprain massage is begun the quicker the recovery. Von Mosengeil's experiments on animals have shown that after massage to injured joints absorption takes place from the articular cavities by means of lymph spaces and small openings communicating with lymphatic vessels, and through these with lymphatic glands. MM. Castex, Fége, Archambault, and others have reported in favour of the early use of massage in dislocations, care being taken not to move nor disturb the joint. Fifteen or twenty days of this treatment is sufficient, and this is just about the length of time required for the repair of the rent in the capsule. M. Castex found that when massage was begun early, or from the very first, in contusions, sprains, and dislocations, not only were the immediate symptoms relieved, but also the subsequent serious consequences that are so apt to follow these injuries. After quoting 1,231 recent cases of sprains treated by massage, A. E. Gallant[7] concludes that this method of treatment of sprained joints will: (1) Prevent swelling, or rapidly disperse it if present; (2) prevent pain, or quickly remove it when due, as it must be, to tension; (3) prevent stiffness and overcome it when already present from disuse; (4) prevent the sense of weakness and restore the part to its original vigour and strength; (5) reduce the time of treatment from weeks to a corresponding number of days; (6) permit the immediate use of the injured member. The only contra-indications mentioned are that massage should never be applied in bacterial inflammations, *i.e.*, tuberculous, gonorrhœal, or pyæmic joints. In two instances of stiffened joints, where the inability to move the limb appeared to arise from rigidity of the tendons and muscular sheaths, Sir B. W. Richardson[8] has injected, subcutaneously, olive oil into the structures, with some success.

M. Lambotte[9] lately operated upon a case of complete dislocation of the iliac bones, with a separation at the symphysis pubis of 10 or 12 cm. The girl, aged seven, had been run over by a vehicle. An immense tear extended from the anterior commissure of the vulva to the coccyx, and there was an incomplete fracture of the right ischio-pubic ramus. The bladder protruded, and the vagina was torn from the right labium majus, the rectum and sphincter being rent for 4 c.m. Two thick silver wires were passed through the pubis, and the bones replaced without difficulty. The rectum was sutured and healed without a sinus. A month after the operation the wire was removed from the symphysis, the cure being perfect and complete.

Two cases of inversion of the limb, or internal rotation in extracapsular fracture of the cervix femoris, are reported by D. M. Greig,[10] in one of which death ensued and a figure is given. The lower fragment, as is usual in these somewhat uncommon cases, was found to lie in front of the upper fragment. Its upper end, being very oblique from above downwards, tended to direct the lower fragment in front of the upper when shortening occurred, and gave rise to the internal rotation. This bears out R. W. Smith's views of this injury. W. A. Lane, again,[11] makes some remarks on the treatment of fractures of the lower extremities, and points out[12] that as regards the mechanical treatment of fractures other than those that are transverse, and in which there is no displacement of fragments, or in which there is little hæmorrhage and swelling, when the broken surfaces can be brought by traction and manipulation into accurate apposition, we must be guided by two important principles: (1) The axes of the fragments must be made to correspond as nearly as possible; and (2) traction must be exerted upon the limb beyond the seat of fracture till the blood and inflammatory material are absorbed sufficiently to allow of the fractured surface being brought down to corresponding levels and into apposition. While he believes that a proportion of fractures of the tibia and fibula occurring in labourers during or after middle life may be treated satisfactorily by extension, regard being had to the symmetrical rotation of the fragments, he is even more convinced than he was that in oblique fractures of both bones occurring somewhat low down, the easiest course is to expose the fractured ends, and, having established continuity of position, to retain them there with screws. As to fractures of the shaft of the femur occurring during the same period of life, he is of opinion that the financial capacity of the individual is depreciated to an extent which is not far, if at all, below that which exists in fractures of the tibia and fibula, and this would appear to be due largely to the imperfect recognition of the two important principles—namely, the symmetrical rotation of fragments, and sufficient traction. Both must be applied in the treatment, either alone being insufficient to obtain a perfect result. D. M. Beddoe[13] gives tables of fractures occurring in working men between the ages of thirty and sixty, as it is for them more particularly that the possession of a firm, stable limb is of such vital importance. He notes the after-results of sixteen cases of fractured femur, and of thirteen cases of Potts' fracture, which he collected without selection. The cases did not extend back more than five years, but even at this period he considered the results as most deplorable, and therefore recommended Lane's method of treatment. Shield[14] sums up Lane's arguments as follows: (1) That changes take place in the joints in the vicinity of a fracture due to the alteration in the direction of transmitted force. (2) That the shortening in a fracture is not due to spasmodic contraction of the muscles so much as to hæmorrhage and effusion. (3) That the immediate treatment of fracture by plaster of Paris is not advisable. (4) That the results of the ordinary treatment of fractures is so bad that operative measures should more widely be adopted. As to the first, he thinks that the trouble to a joint is often greater than the injury itself, and is due to the important fact that joints frequently get severely injured when a bone is fractured. As to the second, he holds that the contrary is the case, seeing the remarkable manner in which great shortening and deformity can be reduced under deep ether anæsthesia. As regards the third point, he agrees that unless for very slight fractures, with either displacement or swelling, the immediate application of plaster of Paris is bad, but its after application to fractures about joints is very valuable. With regard to the fourth point, he asserts that the majority of students and medical men ignore the subject of treatment or give it such scant attention that the ultimate results are too often deplorably bad. If infinite pains and trouble be taken over even oblique fractures, the results are far different to what Lane would lead us to believe, and the results of private practice treatment by careful surgeons would probably show in the vast majority of cases excellent results, without any operative treatment. The practice of keeping the muscles disused and the joints stiff after a fracture is united is responsible for many troubles, while the persistent œdema after fractures is due to venous thrombosis. Gentle daily massage should be commenced at the end of six weeks in the case of an oblique fracture of the leg. Shield has had such good results with the vertical foot-piece, taking care that the great toe, patella, and anterior superior spine are on the same line, that he sees no reason to alter this method. He believes that the operation of screwing together simple fractures should be reserved for a few exceptional very oblique fractures, and always performed by very skilled surgeons, and that if anyone undertook this method without a most thorough practical knowledge of aseptic surgery and of operative proceedings he would probably land his patient and himself in grave disaster. From E. Deanesly's[15] experience at the Wolverhampton General Hospital, where sixty to seventy cases of fracture of the leg are treated every year, he cannot recall a single case that was unable to return to the same employment as before on account of imperfect recovery from the effects of the injury. In a case of fracture of the lower end of the femur, which was broken into three fragments, and the tibia displaced backwards, Walther[16] recently operated with success by open incision into the antero-internal aspect of the joint. The osseous fragments were replaced in their normal relations and fixed by sutures. Twelve months later there was only a little shortening, and the knee slightly swollen. The result was far superior to any other mode of treatment. The joint ould be flexed to a right angle. A review of the ambulant treatment of fractures of the lower extremity adopted by Bardeleben, Korsch, Albers, Krause, and Warbasse, and the results of this method by these authors is given in the "Annals of Surgery."[17] Krause is of opinion that the ambulant treatment must be limited principally to fractures and osteotomies in the region of the malleoli, the leg, and the lower end of the thigh. He does not employ the method in oblique fractures of the femur and fractures of the neck of the same. The basis of the splint is plaster of Paris, re-enforced with braces or supports, in accordance with the taste and ingenuity of the individual surgeon. It allows the patient to rise and walk almost immediately after it is applied. The writer in the "Therapeutic Gazette"[18] thinks that in some simple cases of fractures of the leg and in other selected cases this method has some advantages sufficient to compensate for the added risk which its employment necessarily implies, but that there is no reason why we should depart from our fixation-rest treatment of fractured thigh.

A comparative study of the treatment of fractured patella at the various London hospitals is given by A. Carless.[19] The many different methods of dealing with the fracture indicate the great difference of opinion which exists on the subject. The conflicting views of surgeons as to the necessity or otherwise of suturing the bone or opening the joint in recent fractures are fully described. Carless believes that the "open operation" is preferable in the hands of the antiseptic surgeon to the so-called subcutaneous plans, which almost all involve puncture of the joint. The operative treatment of fractured patella has been the subject of a recent discussion at the New York Surgical Society.[20] G. R. Fowler[21] defers operation until a sufficient time has elapsed to permit the damaged tissues in the neighbourhood to regain their normal condition. He adopts a special method which consists essentially in exposing the fragments as an intermediate proceeding, *i.e.*, after the immediate effects of the injury have subsided, and before the occurrence of ligamentous union, for the purpose of clearing their surfaces of intervening soft parts, and the application of fixation hooks somewhat resembling Malgaigne's, except that a single instead of a double pair is employed. The hooks are permitted to remain for about three weeks. McBurney[22] describes a case of transverse fracture of the patella without separation. The anterior surface was thought to be alone involved, and a groove marked the line of injury from one side to the other. No mobility of any kind existed between the upper and lower ends of the bone. Five weeks later the man had complete power of flexion and extension. H. Lilienthal[23] relates a case treated by massage in a woman aged 28. There was little separation, but crepitus was elicited. A posterior splint was applied, with a figure of eight bandage, and massage of the entire limb begun next day, and repeated twice a day, the fragments being held in position by an assistant. The patient was allowed up on the tenth day, to walk on the eleventh, wearing the bandage. The result was perfect, the patella being only a little longer than its fellow. E. M. Cox[24] sutured with catgut a compound comminuted fracture of the patella in a boy who had fallen from a height of four storeys. There was also fracture of the femur of the same side, and of both bones of the other leg.

There was necrosis of a small fragment, but perfect function resulted. S. F. Forbes[25] reports a successful operation upon a complete dislocation of the knee of nine years' standing. The leg was atrophied, and bent at an angle of 45 degrees. After preparing the surfaces of the bones, he sutured them with six chromatised catgut sutures with one silver ligature; the periosteum was drawn down and sutured with gut, and the external flap treated in the usual manner. The leg was then bandaged with strips of wood up as high as the groin. The silver wire came away at the end of four weeks, and at the end of ten weeks the patient was discharged cured with a straight and useful limb. Maylard and Andrew[26] have met with a very uncommon injury, viz., compound dislocation of the internal cuneiform bone. The bone was replaced, but some gangrene of the skin of the inner side of the sole ensued. This led, after healing had taken place, to contraction on the inner side, so that the great toe came to be nearly at right angle to the foot. The great toe was subsequently amputated.

[7] Med. News, April 30, 1895, and Internat. Med. Mag., June, 1895, p. 388. [8] Med. Record, May, 1895, and Asclepiad. [9] Med. Chronicle, June, 1895, p. 201, and Annals et Bull. de la Soc. de Méd. d'Anvers, March, 1895. [10] Edin. Med. Journal, June, 1895, p. 1,096. [11] Brit. Med. Jour., April 20, 1895. [12] Clinical Journal, April 17, 1895, p. 392. [13] Lancet, June 1, 1895, p. 1,366. [14] Clinical Journal, May 15, 1895, p. 42. [15] Brit. Med. Jour., June 15, 1895, p. 1,319. [16] Rev. de Chir., May, 1895, and Méd. Mod., April 20, 1895. [17] Annals of Surgery, Feb., 1895, p. 187. [18] Ther. Gazette, Feb. 15, 1895. [19] Practitioner, June, 1895, p. 518. [20] Annals of Surgery, May, 1895, p. 602. [21] Annals of Surgery, June, 1895, p. 621. [22] Annals of Surgery, March, 1895, p. 321. [23] Annals of Surgery, May, 1895, p. 602. [24] N.Y. Medical Record, April 27, 1895, p. 538. [25] N.Y. Med. Record, June 8, 1895. [26] Glasgow Med. Journal, June, 1895, p. 461.

